# AII amacrine cells discriminate between heterocellular and homocellular locations when assembling connexin36-containing gap junctions

**DOI:** 10.1242/jcs.133066

**Published:** 2014-03-15

**Authors:** Arndt Meyer, Gerrit Hilgen, Birthe Dorgau, Esther M. Sammler, Reto Weiler, Hannah Monyer, Karin Dedek, Sheriar G. Hormuzdi

**Affiliations:** 1 Research Centre for Neurosensory Sciences/Department of Neurobiology, University of Oldenburg, 26111 Oldenburg, Germany; 2 Division of Neuroscience, University of Dundee, Dundee DD1 9SY, UK; 3 German Cancer Research Center (DKFZ), 69120 Heidelberg, Germany

**Keywords:** Gap junction, Electrical synapse, connexin, Amacrine cell, Bipolar cell, Retina

## Abstract

Electrical synapses (gap junctions) rapidly transmit signals between neurons and are composed of connexins. In neurons, connexin36 (Cx36) is the most abundant isoform; however, the mechanisms underlying formation of Cx36-containing electrical synapses are unknown. We focus on homocellular and heterocellular gap junctions formed by an AII amacrine cell, a key interneuron found in all mammalian retinas. In mice lacking native Cx36 but expressing a variant tagged with enhanced green fluorescent protein at the C-terminus (KO-Cx36-EGFP), heterocellular gap junctions formed between AII cells and ON cone bipolar cells are fully functional, whereas homocellular gap junctions between two AII cells are not formed. A tracer injected into an AII amacrine cell spreads into ON cone bipolar cells but is excluded from other AII cells. Reconstruction of Cx36–EGFP clusters on an AII cell in the KO-Cx36-EGFP genotype confirmed that the number, but not average size, of the clusters is reduced – as expected for AII cells lacking a subset of electrical synapses. Our studies indicate that some neurons exhibit at least two discriminatory mechanisms for assembling Cx36. We suggest that employing different gap-junction-forming mechanisms could provide the means for a cell to regulate its gap junctions in a target-cell-specific manner, even if these junctions contain the same connexin.

## INTRODUCTION

Rapid signal propagation and processing are fundamental to the functioning of neuronal assemblies. Electrical synapses (gap junctions), composed of connexin proteins (Cx), provide a fast means of inter-neuronal communication and are widely distributed in the central nervous system. They serve a variety of important functions, such as neuronal synchronization (Christie et al., 2005), network oscillations (Hormuzdi et al., 2001), lateral and forward transmission (Bloomfield and Völgyi, 2009) and the development of neuronal circuits (Li et al., 2012b; Yu et al., 2012).

Connexins oligomerize into hexameric hemichannels called connexons, and two connexons dock in adjacent membranes to form the intercellular gap junction channel (Kumar and Gilula, 1996). Recent studies suggest that the regulation of electrical synapses might be very complex and dynamic (Flores et al., 2012; Kothmann et al., 2009): gap junctions are most likely to consist of multimolecular components (Li et al., 2012a) in which regulatory, cytoskeletal and scaffolding proteins participate in the regulation of conductance, assembly and turnover.

Connexin36 (Cx36) represents the most widespread neuronal connexin and is expressed in a variety of brain regions, including the cortex, hippocampus, inferior olive, olfactory bulb, cerebellum and retina (Söhl et al., 2005). Although Cx36 is known to interact with ZO-1, ZO-2 and other scaffolding proteins (Ciolofan et al., 2006; Li et al., 2008; Li et al., 2012a), the mechanisms used to assemble Cx36 into functional gap junctions are largely unknown. Helbig and colleagues (Helbig et al., 2010) have provided evidence that an intact C-terminus is needed to target Cx36 to the plasma membrane. Here, we extended our studies on the assembly of Cx36 tagged with enhanced green fluorescent protein (Cx36–EGFP) in the presence and absence of the native Cx36. We used the mouse retina as a model system because Cx36 is expressed in many different retinal neurons, including photoreceptors and several amacrine, bipolar and ganglion cell types (Bloomfield and Völgyi, 2009; Deans et al., 2002; Feigenspan et al., 2004; Güldenagel et al., 2001; Han and Massey, 2005; Lin et al., 2005; Pan et al., 2010; Schubert et al., 2005).

**Fig. 1.**
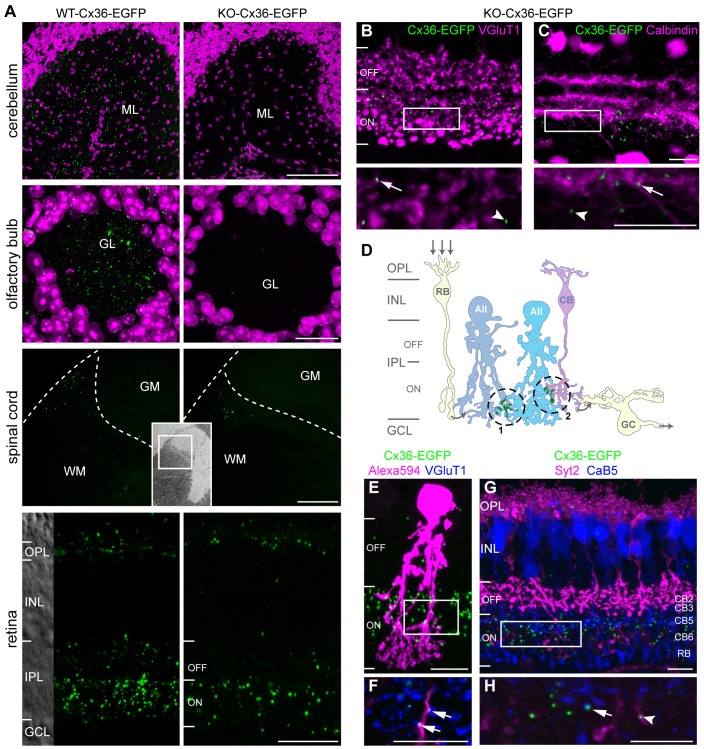
**AII cells are able to form Cx36–EGFP gap junctions in the KO-Cx36-EGFP retina.** (A) Comparison of EGFP clusters in the molecular layer of the cerebellum (ML), olfactory bulb glomeruli (GL), spinal cord (WM and GM, white and gray matter, respectively. The inset image shows an overview of the spinal cord; the white square indicates the magnified area shown in the two images.) and outer and inner plexiform layer (OPL and IPL, respectively) of the retina of WT-Cx36-EGFP or KO-Cx36-EGFP littermates. Neurons in the cerebellum or olfactory bulb lack Cx36–EGFP gap junctions in the KO-Cx36-EGFP, whereas some neurons in the spinal cord and retinal neurons in the IPL establish junctions, even when the native Cx36 is lacking. (B,C) In the IPL of the KO-Cx36-EGFP retina, Cx36–EGFP clusters (green) partially colocalized with labeling for VGluT1 (magenta; B), a marker for bipolar cell terminals; and calbindin (magenta; C), a marker for various amacrine and ganglion cell types. Arrows and arrowheads point to clusters colocalizing and not colocalizing with the marker, respectively. The boxed regions in B and C are magnified in the respective panels below. (D) A schematic showing Cx36 gap junctions (green) formed between two AII amacrine cells (AII; dashed circle 1) and between an AII and an ON cone bipolar cell (CB; dashed circle 2). The arrows illustrate the flow of light-evoked signals from rod bipolar cells (RB) to ganglion cells (GC) through heterocellular gap junctions linking AII and ON cone bipolar cells. (E,F) An Alexa-Fluor-594-injected AII cell (magenta, boxed region in E is magnified in the single scan shown in F) from a KO-Cx36-EGFP mouse contained Cx36–EGFP clusters (green, arrows in F), which also colocalized with VGluT1 (blue, F). (G,H) Antibodies against synaptotagmin2 (SYT2: magenta, G,H; the boxed region in G is magnified in the single scan shown in H) and calcium-binding protein 5 (CAB5: blue, G,H) identify additional cone bipolar subtypes; the lamination of their terminal processes (the cone bipolar type is indicated on the right) determines type 5 (arrow in H) and 6 (arrowhead in H) as subtypes that form gap junctions with AII cells. ON and OFF sublayers are indicated. Scale bars: A, cerebellum and spinal cord, 100 µm; olfactory bulb and retina, 20 µm; C–F, 10 µm.

One of the Cx36-expressing cells is the AII amacrine cell (AII), the most abundant amacrine cell in the mammalian retina. Like several other retinal neurons (cones, ganglion cells), AII cells form two classes of Cx36-containing electrical synapses. Homocellular gap junctions (Fig. 1D, circle 1), present between adjacent AII cells, are composed entirely of Cx36 and convert the AII population into an electrically coupled network (Mills and Massey, 1995; Vaney, 1991; Veruki and Hartveit, 2002) important for signal-to-noise optimization (Dunn et al., 2006). Heterocellular gap junctions, established between AII cells and ON cone bipolar cells (Fig. 1D, circle 2), comprise Cx36 and perhaps bihomotypic (Li et al., 2008) or heterotypic Cx36–Cx45 complexes (Dedek et al., 2006). In low light levels, rod signals transmitted to AII cells are relayed via these gap junctions onto ON cone bipolar cells and thence to ganglion cells. Thus, Cx36-containing electrical synapses play a crucial role in funneling rod signals into cone pathways and, as a consequence, Cx36 knockout mice have impaired rod vision (Deans et al., 2002; Güldenagel et al., 2001). As the two classes of AII cell gap junctions perform different functions, it is not surprising that they are modulated differently (Mills and Massey, 1995), possibly by phosphorylation of Cx36 (Kothmann et al., 2009). A structural dissimilarity is also suggested by electron microscopy analysis, which identifies a deposit in the AII cell, exclusively underneath the heterocellular junction (Anderson et al., 2011). Thus, the two classes of AII cell gap junctions subserve different functions, are ultrastructurally different and are regulated differently, even though the cell contributes Cx36 connexons to both.

Here, we show that the mechanisms used to assemble Cx36 differ between homocellular and heterocellular junctions in AII cells. This demonstrates for the first time that multiple pathways for assembling a single connexin into gap junctions exist in a single neuron and, furthermore, that these mechanisms assemble distinct gap junctions that are discernible by the identity of the synaptic partner.

## RESULTS

For this study, several different mouse lines were used: (1) a transgenic line (formerly designated TgCx36-EGFP) (Christie et al., 2005; Helbig et al., 2010), which expresses full-length Cx36 with EGFP appended to the carboxy terminus and also the native Cx36. This mouse line is referred to as WT-Cx36-EGFP throughout the manuscript. (2) A transgenic line, which only expresses the tagged Cx36 protein and lacks the wild-type Cx36 protein, here designated as KO-Cx36-EGFP. (3) A mouse line expressing the tagged protein and heterozygous for the native Cx36 (HET-Cx36-EGFP). Results were compared with Cx36-expressing and Cx36-deficient mice from the same background [wild type (WT) and knockout (KO), respectively].

### The Cx36–EGFP fusion protein assembles into a subset of gap junctions in the KO-Cx36-EGFP retina

The WT-Cx36-EGFP mouse line has provided some clues to the mechanism of electrical synapse formation. Electrical synapses in the transgenic brain, which are visible as distinct fluorescent clusters, contain the Cx36–EGFP protein but the fusion protein will only assemble into gap junctions if neurons also express an unaltered genomic copy of Cx36, presumably because an intact carboxy terminus is required for correct targeting or assembly (Christie et al., 2005; Helbig et al., 2010). Thus, puncta are lacking in the cerebellum and olfactory bulb of KO-Cx36-EGFP mice but not in WT-Cx36-EGFP mice (Fig. 1A). Surprisingly, a substantial, though reduced, number of fluorescent clusters persist in the white matter of the spinal cord and the plexiform layers of the retina of KO-Cx36-EGFP mice (Fig. 1A), suggesting that neurons of the central nervous system possess distinct mechanisms for the assembly of Cx36. We examined this possibility further and focused on the retina.

We first evaluated the clusters of EGFP formed by the WT-Cx36-EGFP genotype in the ON sublayer of the inner plexiform layer (IPL), which contains the majority of Cx36-containing gap junctions in the IPL. Immunostaining for Cx36 demonstrated that most EGFP clusters contained Cx36 [95%±2 (±s.e.m.); *n* = 2 mice per genotype] and that very few Cx36-positive clusters lacked EGFP (7%±1). The density of Cx36-immunoreactive plaques in WT-Cx36-EGFP mice (0.272 plaques/µm^2^±0.011) was similar to that formed in the WT (0.297 plaques/µm^2^±0.011). Thus, the number of Cx36-containing gap junctions in the ON IPL is not influenced by the presence of the Cx36–EGFP protein, and WT-Cx36-EGFP mice contained a complete wild-type set of gap junctions. In comparison with WT-Cx36-EGFP (0.178/µm^2^±0.033), the KO-Cx36-EGFP ON IPL contained significantly fewer EGFP clusters (0.043/µm^2^±0.007; *n* = 4 mice per genotype; *P* = 0.0077), indicating that a smaller set of Cx36-containing gap junctions (∼24%) is present in this genotype (Fig. 1A, right column).

### Functional heterocellular gap junctions between AII and ON cone bipolar cells are formed in the absence of the wild-type Cx36 protein

In the ON IPL, bipolar, amacrine and ganglion cells have been described to form gap junctions containing Cx36 (Bloomfield and Völgyi, 2009). Labeling of the KO-Cx36-EGFP retina for vesicular glutamate transporter 1 (VGluT1) and calbindin revealed that Cx36–EGFP puncta are located on VGluT1-positive terminals of bipolar cells (Fig. 1B) and calbindin-positive processes of amacrine or ganglion cells (Fig. 1C). EGFP-containing gap junctions found in the ON IPL could also be formed by calbindin-negative AII cells, which represent the most numerous amacrine cell in the mouse retina (Demb and Singer, 2012). These gap junctions can be subdivided into those that mediate coupling between AII cells (homocellular) or between AII and ON cone bipolar cells (heterocellular) (Fig. 1D). We determined whether these two classes of gap junction might be retained in the KO-Cx36-EGFP mouse (Fig. 1E–H). The distal dendrites of identified AII cells, visualized after injecting Alexa Fluor 594, were observed to colocalize with numerous EGFP puncta (Fig. 1E,F), some of which also colocalized with VGluT1 (Fig. 1F), a marker for all bipolar-cell terminals. Puncta were also found to colocalize with terminals positive for calcium-binding protein 5 (CABP5, herein referred to as CAB5) or synaptotagmin-2 (SYT2) (Fig. 1G,H), marking type 5 or type 6 ON cone bipolar cells, respectively. Thus, in KO-Cx36-EGFP mice, AII cells and ON cone bipolar subtypes known to form Cx36-containing gap junctions in the wild type (Dedek et al., 2006) can replace Cx36 with the fusion variant.

**Fig. 2.**
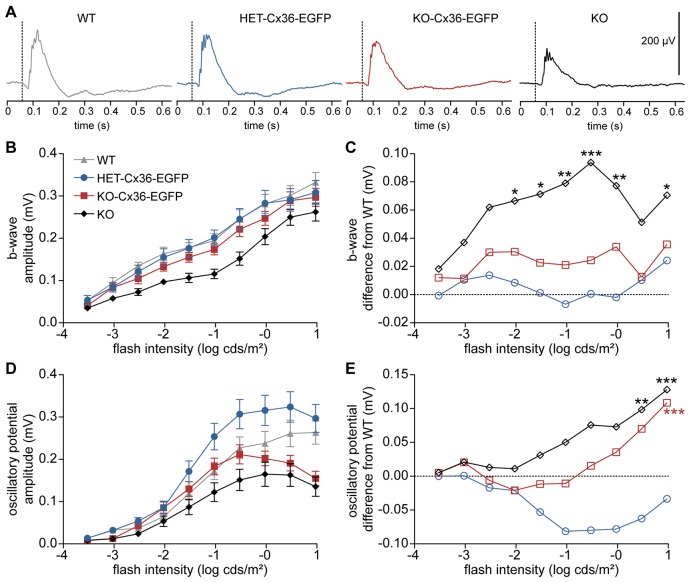
**Electroretinograms reveal that heterocellular gap junctions between AII and cone bipolar cells function normally in the KO-Cx36-EGFP retina.** (A) The mean dark-adapted ERG responses of WT, HET-Cx36-EGFP, KO-Cx36-EGFP or KO mice, respectively, at a light intensity of −1.5 log cds/m^2^ are shown. (B) Scotopic intensity response curves for b-waves of the different genotypes (*n* = 10 mice each). (C) b-wave amplitude differences to WT are plotted for HET-Cx36-EGFP, KO-Cx36-EGFP or KO mice. KO mice had significantly smaller b-wave amplitudes than WT. Although b-wave amplitudes of KO-Cx36-EGFP mice were generally smaller than WT (two-way ANOVA, *P* = 0.0013), post hoc analysis did not reveal significantly different means. (D,E) By contrast, oscillatory potentials (D) were smaller in both KO and KO-Cx36-EGFP mice than in the WT. Differences to the WT (E) were statistically significant at the two highest intensities. Two-way ANOVA for repeated measurements with post hoc Bonferroni test: *P<0.05; **P<0.01; ***P<0.001.

AII cells are central to the primary rod pathway wherein rod signals are transmitted from AII cells to ON cone bipolar cells through Cx36-containing gap junctions. Scotopic (low light) vision thus relies on these heterocellular gap junctions and can be monitored by electroretinography as the positive b-wave of the electroretinogram (ERG) mostly arises from ON (rod and cone) bipolar cells (Weymouth and Vingrys, 2008). As expected (Güldenagel et al., 2001), scotopic b-wave amplitudes (Fig. 2) were significantly smaller in KO mice when compared with the WT (Fig. 2A–C). At low light levels, this can be attributed to the impaired primary rod pathway, and at higher light intensities this might be due to impaired rod–cone coupling (Abd-El-Barr et al., 2009) as Cx36 also represents the connexin on the cone side of the gap junction (Feigenspan et al., 2004).

Amplitudes in KO-Cx36-EGFP mice were larger than in KO mice but generally smaller than in WT mice [two-way analysis of variance (ANOVA), *P* = 0.0013]. However, post hoc analysis indicated that means were no different from WT at all light intensities tested (Fig. 2C), consistent with a previous study (Feigenspan et al., 2004). This suggests that gap junctions between AII and ON cone bipolar cells, as well as between rods and cones, are present and functional in KO-Cx36-EGFP mice. Interestingly, oscillatory potentials, which are thought to originate from amacrine cell circuits in the inner retina (Dong et al., 2004; Wachtmeister, 1998), were significantly diminished in KO and KO-Cx36-EGFP mice at higher light intensities (Fig. 2D,E). As oscillatory potentials are largest at mesopic light levels (Wachtmeister, 1998), one might speculate that any effect of deficiency of Cx36 only becomes visible when oscillatory potentials are at their maximum. Thus, our data point to a differential effect on amacrine cell circuits in mice expressing only the Cx36–EGFP fusion protein (KO-Cx36-EGFP).

**Fig. 3.**
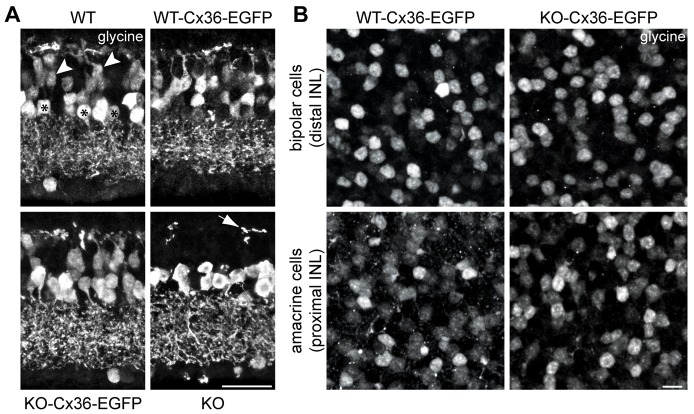
**Heterocellular gap junctions between AII and ON cone bipolar cells in the KO-Cx36-EGFP retina show normal neurotransmitter coupling.** (A) Collapsed confocal stacks demonstrate that only bipolar cells in the KO mice lack glycine immunoreactivity (arrowheads and asterisks indicate the locations of glycine-positive ON cone bipolar and amacrine cell layers, respectively; the arrow in the KO points to putative interplexiform dendrites of glycinergic amacrine cells that become visible because ON cone bipolar cells lack glycine in this genotype). (B) Confocal stacks comparing glycine immunoreactivity in the proximal and distal inner nuclear layers (INL; representing the amacrine and bipolar-cell layers, respectively) of HET-Cx36-EGFP (an example from WT-Cx36-EGFP is shown) and KO-Cx36-EGFP retina. Quantification demonstrated that the number of glycine-positive cells in the proximal INL (HET-Cx36-EGFP = 66±2/0.01 mm^2^; KO-Cx36-EGFP = 64±3/0.01 mm^2^; *n* = 2 mice per genotype, 3–4 scans per mouse; *P* = 0.503) and distal INL (HET-Cx36-EGFP = 65±1/0.01 mm^2^; KO-Cx36-EGFP = 67±3/0.01 mm^2^; *P* = 0.57) was the same in both genotypes. To determine whether all ON cone bipolar cell types were represented in the glycine-positive population, the ratio between glycine-positive cells in the distal and proximal INL was calculated. The ratio was similar (HET-Cx36-EGFP = 1±0.02; KO-Cx36-EGFP = 1.06±0.05; *P* = 0.368), suggesting that equal numbers of ON cone bipolar cells and thus the same ON cone bipolar subtypes are able to form gap junctions with AII amacrine cells in the two genotypes. Scale bars: A, 20 µm; B, 10 µm.

The presence of intact heterocellular gap junctions in KO-Cx36-EGFP mice was confirmed by immunostaining for glycine (Fig. 3). AII cells transmit glycine to ON cone bipolar cells via these junctions (Deans et al., 2002; Güldenagel et al., 2001), and thus ON cone bipolar cells in the KO mice are devoid of glycine (Fig. 3A). By contrast, glycine-positive cells were present in amacrine and bipolar-cell layers in controls (WT and WT-Cx36-EGFP) and in KO-Cx36-EGFP mice, indicating that the transfer of glycine from AII to ON cone bipolar cells via heterocellular gap junctions was unimpaired in these retinas. We further quantified this to rule out that a certain bipolar-cell class, for example the Cx36-expressing type 7 ON cone bipolar cells (Han and Massey, 2005; Lin et al., 2005), is lacking from the glycine-positive cells in KO-Cx36-EGFP. Thus, we counted the number of glycine-positive cells in the amacrine- or bipolar-cell layer of whole-mount retinal preparations obtained from HET-Cx36-EGFP and KO-Cx36-EGFP mice (Fig. 3B). The number of glycine-positive cells in the amacrine- or bipolar-cell layer was not significantly different between genotypes (for numbers, see Fig. 3 legend), ruling out the possibility that the presence and/or absence of heterocellular gap junctions varied with the bipolar-cell class (Fig. 3B). Overall, our data show that heterocellular gap junctions are functionally intact in the KO-Cx36-EGFP retina and are similar to those of the WT.

### A specific loss of homocellular AII–AII gap junctions is revealed by tracer coupling

**Fig. 4.**
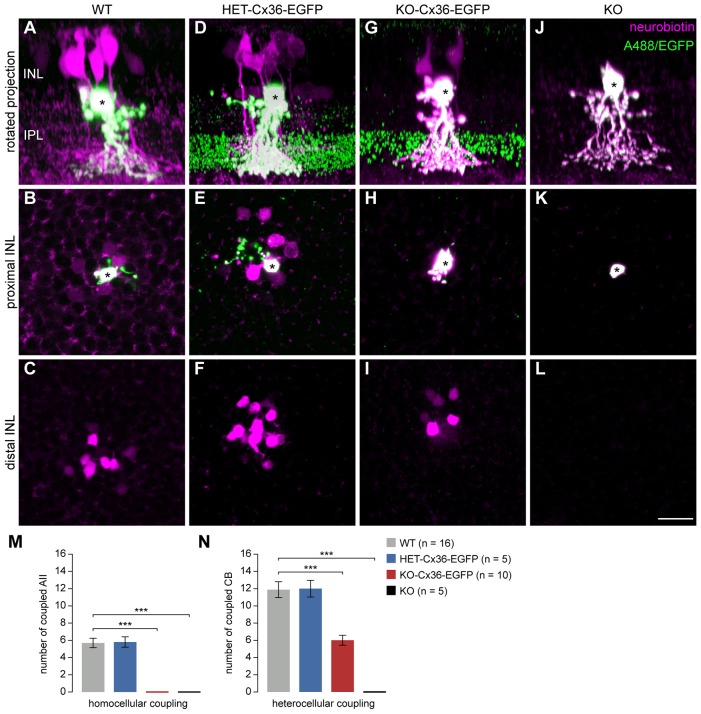
**Homocellular AII–AII coupling is disrupted in the KO-Cx36-EGFP retina.** Example results of AII cell tracer-coupling experiments (asterisks represent Alexa-Fluor-488-injected AII cells; the tracer neurobiotin is shown in magenta). (A,D,G,J) Rotated projections showing the injected AII (green), Cx36–EGFP clusters (green), and neurobiotin-containing coupled cells (magenta). (B,E,H,K) and (C,F,I,L) collapsed confocal stacks derived from the proximal and distal INL, respectively. AII cells in the KO-Cx36-EGFP retina have functional heterocellular gap junctions between AII and ON cone bipolar cells but lack homocellular AII–AII gap junctions (H,I). Dye-injected AII cells in KO-Cx36-EGFP (G) and KO mice (J) contain higher amounts of neurobiotin as they lack homocellular (KO and KO-Cx36-EGFP) and heterocellular gap junctions (KO). This makes the outline of these cells appear in magenta as opposed to green (due to the co-injected Alexa Fluor 488) for injected AII cells in the WT and HET-Cx36-EGFP genotypes. (M,N) Graph indicating the number of AII cells (M) and ON cone bipolar (CB) cells (N) coupled to the injected AII cell. Homocellular AII–AII gap junctions are completely lacking in the KO-Cx36-EGFP retina. The reduction in the number of coupled bipolar cells in this genotype could be attributed to the absence of homocellular coupling (see Fig. 5). A *t*-test was used to compare means with the mean in the WT: ***P<0.001. Scale bar: 20 µm.

As ERG recordings and glycine staining tested only the functionality of the pathway between AII and ON cone bipolar cells without a direct examination of the gap junctions involved, we assessed the homocellular and heterocellular gap junctions of AII cells by injection of the tracer neurobiotin (Fig. 4). The spread of tracer from a dye-injected AII cell (marked by a coinjected, gap-junction-impermeant dye, Alexa Fluor 488) into other AII and ON cone bipolar cells is only possible when homocellular and heterocellular gap junctions, respectively, are present. Our experiments demonstrated that the transfer of tracer into coupled cells in the homo- and hetero-cellular networks was comparable for WT (Fig. 4A–C,M,N) and HET-Cx36-EGFP (Fig. 4D–F,M,N), but lacking in KO mice (Fig. 4J–L,M,N). Surprisingly, homocellular coupling between AII cells was completely lacking in the retinas from KO-Cx36-EGFP mice (Fig. 4G,H,M), despite the persistence of heterocellular coupling between AII and ON-cone bipolar cells (Fig. 4I,N); a coupled AII cell was not identified in this genotype, whereas zero coupling was never observed in WT and HET-Cx36-EGFP mice. Thus, our results suggest that the KO-Cx36-EGFP retina has a selective loss of gap junctions between AII cells resulting in a network of AII cells that is able to transfer tracer into ON cone bipolar cells only.

**Fig. 5.**
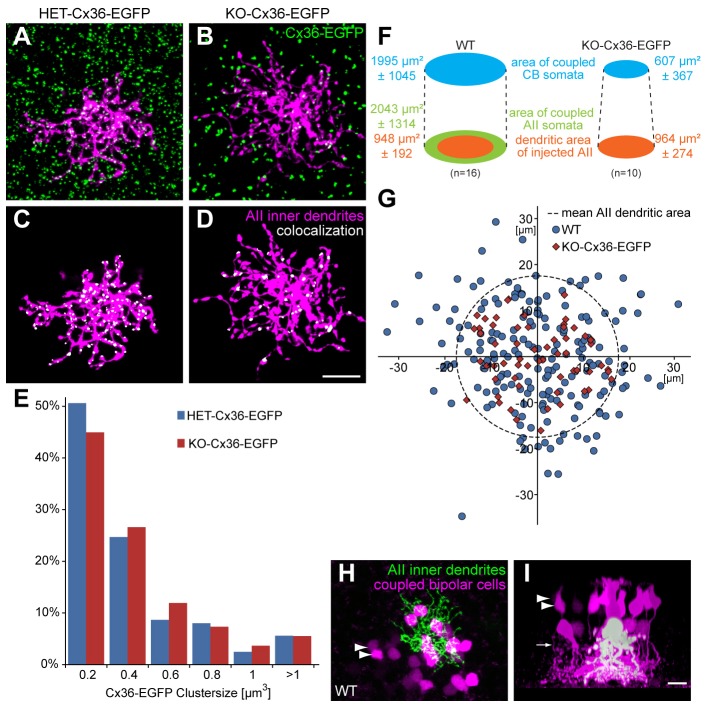
**Heterocellular gap junctions formed in the KO-Cx36-EGFP retina are similar to the wild type in size and allow a spread of tracer comparable to that in the wild type.** (A–E) The inner dendrites of a dye-injected AII cell (magenta) in the HET-Cx36-EGFP (A) and KO-Cx36-EGFP retina (B) demonstrating the distribution of Cx36–EGFP clusters (green) in the vicinity. Clusters that colocalized with AII cell dendrites (C,D, white) were identified and distributed into discrete size classes (E). Although the number of Cx36-EGFP clusters was reduced in KO-Cx36-EGFP mice, clusters were similar in size in both genotypes. Thus, the absence of genomic Cx36 did not impede cluster formation in the KO-Cx36-EGFP retina. (F) The mean±s.e.m. of the total area covered by coupled ON cone bipolar (CB) cells, coupled AII cells and dendrites of an injected AII cell from WT and KO-Cx36-EGFP. (G) Spatial distribution of coupled ON cone bipolar cells relative to the area occupied by the injected AII dendrites for WT and KO-Cx36-EGFP. For the KO-Cx36-EGFP mouse, all coupled ON cone bipolar cells are situated within the dendritic field of the injected AII cell (*n* = 60, 10 injections), but, for the WT, a substantial number are located outside (*n* = 53, 16 injections). This distribution reflects the tracer-spread within the AII layer, which for the WT extends to all coupled AII but is restricted to the injected cell for the KO-Cx36-EGFP. (H,I) Two views of the same confocal stack taken from a WT mouse. The distribution of coupled ON cone bipolar somata (magenta) and injected AII dendrites (green, left; white, right) demonstrates occasional tracer-containing ON cone bipolar cells (arrowheads in H,I) whose axons are clearly visible (arrow in I) and considerably distant from the injected AII. This validates that in the WT, the tracer spreads to ON cone bipolar cells from uninjected but coupled AII cells. Scale bars: 10 µm.

The number of ON cone bipolar cells containing the tracer in the KO-Cx36-EGFP retina was significantly reduced in comparison with that of the WT retina (Fig. 4N, WT: 11.88±0.92, *n* = 16; KO-Cx36-EGFP: 6±0.56, *n* = 10; *P* = 0.0001). One potential explanation is that the tracer was unable to spread into selective classes of bipolar cells. However, quantitative analysis of immunoreactivity for glycine (Fig. 3A,B) showed this not to be the case. The possibility that gap junctions assembled from Cx36–EGFP have a reduced permeability or open probability is contradicted by previous *in vitro* studies showing that intercellular channels made of Cx36–EGFP have a conductance similar to that of Cx36 channels (Helbig et al., 2010). Another possibility is that the assembly of Cx36–EGFP into heterocellular gap junctions in the KO-Cx36-EGFP genotype might take place inefficiently, resulting in diminished tracer coupling. In this event, one would expect that the number of connexons inserted into the plaque is reduced and smaller gap junctions are formed. To examine this possibility, we compared the number and size of EGFP clusters present on dye-injected AII cells in retinas of KO-Cx36-EGFP or HET-Cx36-EGFP mice (Fig. 5A–E). The number of clusters located on a dye-injected AII cell was significantly decreased in KO-Cx36-EGFP mice (KO-Cx36-EGFP = 30 clusters ±6, *n* = 9; HET-Cx36-EGFP = 169 clusters ±13, *n* = 9; *P* = 6×10^−9^) consistent with the loss of coupling within the network of AII cells. Although Cx36–EGFP clusters tended to be slightly larger in the KO-Cx36-EGFP genotype, cluster size was not significantly different between the genotypes (Fig. 5E; KO-Cx36-EGFP median = 0.23 µm^3^, *n* = 109 clusters; WT-Cx36-EGFP median = 0.20 µm^3^, *n* = 162 clusters; *P* = 0.1576). A similar observation was made when all Cx36–EGFP clusters of the ON layer were compared (data not shown). Thus, the fusion protein is not impeded in its ability to traffic to, and assemble into, the synapse and is efficiently incorporated into the heterocellular junction.

It is possible that the absence of homocellular AII–AII coupling influenced our results by preventing the inclusion (in the KO-Cx36-EGFP dataset) of all ON cone bipolar cells that would normally receive tracer from uninjected but coupled AII cells (Mills and Massey, 1995). The validity of this assertion for our data is evident from Fig. 5F–I. The distribution of ON cone bipolar cells containing the tracer is much greater in WT cells (Fig. 5F,G), even though the average dendritic fields of AII cells injected with the tracer in the WT and KO-Cx36-EGFP genotypes are very similar. Spread of the tracer in ON cone bipolar cells of the KO-Cx36-EGFP mice is limited to the dendritic area of the injected AII cell, but, in WT retinas, the area reflects the much larger total area of tracer spread in the AII layer (Fig. 5F,G). Finally, in some injections of the WT retinas (Fig. 5H,I), we occasionally identified well-filled tracer-containing ON cone bipolar cells that were too remote from the injected AII cell to make contact, suggesting that they received the tracer from one or more AII cells homocellularly-coupled to the injected AII cell. In conclusion, we attribute the observed reduction in the number of bipolar cells containing the dye in the KO-Cx36-EGFP mouse (Fig. 4N) to the absence of secondary dye-spread from coupled AII cells (Mills and Massey, 1995) and not to any deficiency in the number and/or function of heterocellular gap junctions.

**Fig. 6.**
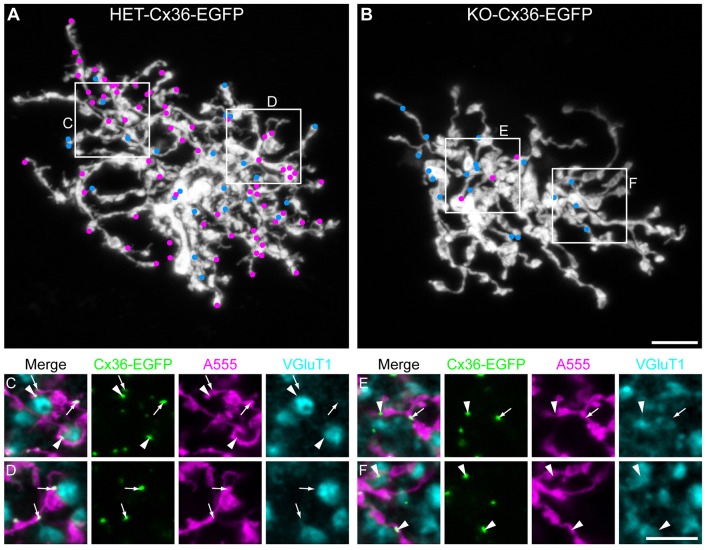
**The majority of AII cell EGFP clusters in the KO-Cx36-EGFP retina colocalize with bipolar cell terminals.** Alexa-Fluor-555-injected AII cells in the HET-Cx36-EGFP (A,C,D) and KO-Cx36-EGFP retina (B,E,F) were immunolabeled for the bipolar-cell synaptic marker VGluT1. EGFP clusters (blue and magenta dots) colocalizing with the inner AII dendrites (white) are represented in A and B; blue and magenta dots represent clusters that do and do not colocalize with VGluT1 immunoreactivity, respectively. (C–F) Substacks of thickness 1–1.5 µm from the boxes indicated in A and B showing Cx36–EGFP clusters (green) colocalizing only with the injected cell (magenta, arrows) or additionally colocalizing with VGluT1 (cyan, arrowheads). Whereas puncta that are localized on the AII cell but lack VGLuT1 immunoreactivity predominate in the HET-Cx36-EGFP retina, such puncta are significantly fewer in the KO-Cx36-EGFP retina. Scale bars: 5 µm.

The above data are consistent with the loss of AII–AII coupling due to the absence of homocellular gap junctions. In order to confirm that AII cells only form heterocellular gap junctions in the ON IPL of KO-Cx36-EGFP mice, we determined, for both genotypes, the fraction of all Cx36–EGFP clusters present on the processes of dye-injected AII cells that colocalize with opposing VGluT1-containing terminals (representing the axon terminals of ON bipolar cells; Fig. 6). The number of clusters formed on injected AII cells in the HET-Cx36-EGFP and KO-Cx36-EGFP was similar to that reported above (142 clusters ±17 and 27±3, respectively), whereas the fraction that colocalized with VGluT1 terminals was significantly different (45.3%±8.1 and 81.3%±7.9, respectively, *n* = 13 injected AII cells each, *P* = 0.00428) and considerably larger for the KO-Cx36-EGFP retina. These findings support the conclusion that AII-AII gap junctions do not form in the KO-Cx36-EGFP ON IPL, thereby accounting for the absence of tracer coupling in this network.

The significantly diminished number of Cx36–EGFP clusters formed by AII cells in the KO-Cx36-EGFP retina (Fig. 5D) in comparison with WT-Cx36-EGFP (Fig. 5C) therefore implies that only a minority of AII cell gap junctions are involved in heterocellular coupling (∼30 of 170 clusters). Two independent methods enable us to estimate the fraction of heterocellular gap junctions between AII and ON cone bipolar cells among all Cx36–EGFP plaques in the ON IPL of the KO-Cx36-EGFP retina. First, we multiplied the number of Cx36–EGFP clusters present on the dendrites of a dye-injected AII cell (*n* = 9 cells) by the expected number of AII cells in the scanned area (2583 AII cells/mm^2^) (Rice and Curran, 2000) to give an estimate of the total number of heterocellular gap junctions in the scanned region. This number was then divided by the total number of Cx36–EGFP plaques observed in the acquired volume. Based on these calculations, we estimated the fraction of heterocellular gap junctions between AII and ON cone bipolar cells among all Cx36–EGFP plaques in the ON IPL of the KO-Cx36-EGFP retina to be 55%±8. The second estimate was based on staining vertical sections with a marker for all bipolar-cell terminals, VGluT1 (Fig. 1B). As Cx36 is not expressed in rod bipolar cells (Feigenspan et al., 2004), the overlap of Cx36–EGFP and VGluT1 in the ON IPL of KO-Cx36-EGFP mice most likely reflects gap junctions between AII and ON cone bipolar cells. We calculated this amount of colocalization to be 47%±4 (*n* = 3 mice; Fig. 1B). Thus, the two independent estimates gave very similar results and suggest that ∼50% of all Cx36–EGFP clusters in the ON IPL of KO-Cx36-EGFP mice contribute to gap junctions between AII and cone bipolar cells. The remaining ∼50% are most likely contributed by other amacrine and ganglion cells (Fig. 1C) as multiple subtypes of amacrine and ganglion cells have been shown to form gap junctions comprising Cx36 (Pan et al., 2010).

### Interaction with ZO-1 or Cx45 does not enable Cx36–EGFP to assemble specifically into heterocellular gap junctions

**Fig. 7.**
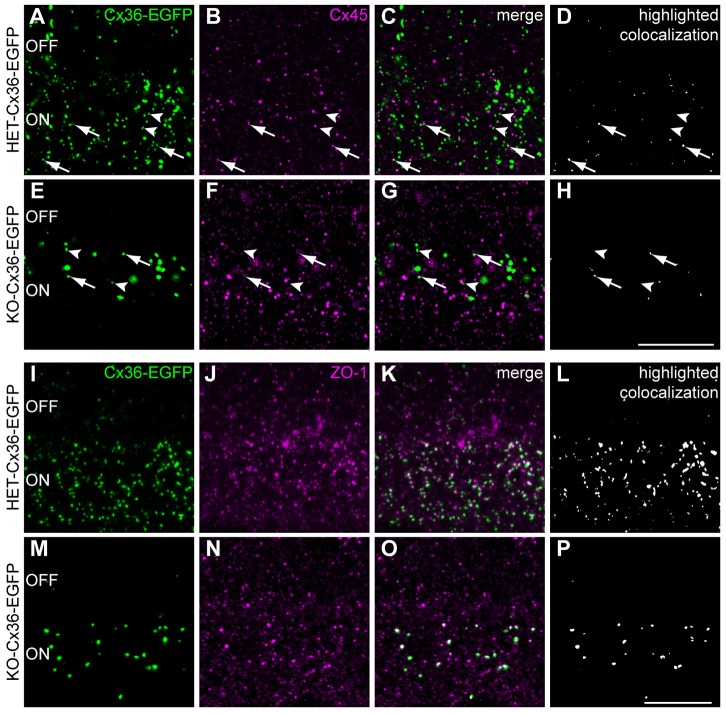
**The possible association of Cx36-EGFP with ZO-1 or Cx45 does not explain its ability to assemble into gap junctions in the KO-Cx36-EGFP retina.** (A–H) Double labeling of Cx36–EGFP (green, A,E) and Cx45 (magenta, B,F) demonstrates only a few colocalized puncta (C,G) in the ON IPL of HET-Cx36-EGFP (A-D) and KO-Cx36-EGFP retinas (E-H). Colocalized pixels are highlighted in white in D and H. Arrows indicate colocalized clusters whereas arrowheads show some clusters lacking colocalization with Cx45. For numbers, please refer to the Results section. (I-P) A similar analysis to that in A–H indicated that the majority of Cx36–EGFP puncta in both genotypes colocalized with staining by an antibody against ZO-1 (magenta, J,N; for numbers, please refer to the Results section). Thus, proximity to and/or coassembly with ZO-1 is not sufficient for the assembly of Cx36–EGFP whereas colocalization with Cx45 is not necessary for the formation of gap junctions exclusively comprising Cx36–EGFP. ON and OFF sublayers are indicated. Scale bars: 10 µm.

Cx36 associates with the scaffold protein ZO-1 (Ciolofan et al., 2006; Li et al., 2004a; Li et al., 2004b; Rash et al., 2004). A recent study has suggested that ZO-1 provides co-scaffolding for Cx36 and Cx45, leading to their deposition into retinal gap junctions to form bihomotypic intercellular channels (Li et al., 2008). Moreover, heterocellular gap junctions between AII and ON cone bipolar cells might contain heterotypic channels arising through the contribution of Cx36-containing connexons by AII and Cx45-containing connexons by ON cone bipolar cells (Dedek et al., 2006). Therefore, association with either or both ZO-1 and Cx45 might enable Cx36–EGFP to assemble into heterocellular gap junctions, independently of the wild-type Cx36 protein. To investigate this, we examined the distribution of ZO-1 and Cx45, relative to Cx36–EGFP fluorescent puncta (Fig. 7). Quantification (*n* = 4 animals per genotype) indicated that only 12%±2 (HET-Cx36-EGFP) and 12%±3 (KO-Cx36-EGFP) of Cx36–EGFP puncta colocalized with immunoreactivity for Cx45 in the ON IPL (Fig. 7A–H). Thus, many gap junctions assemble from Cx36–EGFP, even without the possible influence of Cx45. Also, the observed overlap between Cx36 and Cx45 (12%±3) is far below our estimate of the number of gap junctions involved in heterocellular coupling between AII and ON cone bipolar cells in the KO-Cx36-EGFP retina (50%).

Co-labeling of retinas with ZO-1 (Fig. 7I–P) revealed that the majority of Cx36–EGFP puncta in both genotypes colocalized with ZO-1 (HET-Cx36-EGFP: 78%±4; KO-Cx36-EGFP: 81%±9; *n* = 3 mice per genotype; *P* = 0.819). The extensive overlap (∼81%) indicates that many Cx36–EGFP clusters that are colocalized with ZO-1 in the ON IPL of the HET-Cx36-EGFP mouse do not assemble in the absence of the wild-type Cx36 as the ON IPL of KO-Cx36-EGFP contains only 24% of the Cx36–EGFP clusters found in the HET-Cx36-EGFP retina. We therefore suggest that proximity to and/or co-assembly with Cx45 or ZO-1 cannot account for the formation of gap junctions comprising exclusively Cx36–EGFP.

## DISCUSSION

### Two discriminatory mechanisms establish AII amacrine cell gap junctions

**Fig. 8.**
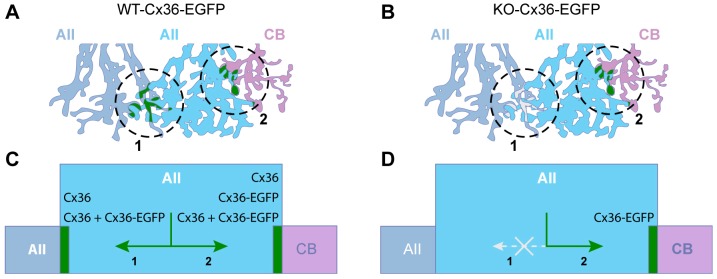
**Two distinct pathways exist for the assembly of Cx36 into AII gap junctions.** A cellular (A,B) and molecular perspective (C,D) showing Cx36–EGFP gap junctions (A, green) formed between two AII cells (A, circle 1; C, arrow 1) and between an AII and an ON cone bipolar cell (CB; A, circle 2; C, arrow 2). (C,D) Different combinations of Cx36 variants are predicted to assemble into AII gap junctions in WT-Cx36-EGFP (C) and KO-Cx36-EGFP mice (D). Both types of gap junctions contain Cx36–EGFP in WT-Cx36-EGFP mice (C); however, only heterocellular gap junctions contain Cx36–EGFP in the KO-Cx36-EGFP mouse (D); the homocellular AII–AII gap junction does not form in this genotype. In summary, AII amacrine cells utilize at least two distinct mechanisms that target Cx36 for assembly; mechanism 1 operates for homocellular assembly and requires that complexes contain the native Cx36, whereas mechanism 2 operates only at the heterocellular synapse and can even assemble complexes comprising solely Cx36–EGFP.

In this study, we show that the incorporation of Cx36–EGFP into homocellular AII–AII gap junctions requires wild-type Cx36 expressed from the genomic allele, whereas Cx36–EGFP assembly into heterocellular gap junctions between AII and ON cone bipolar cells does not (Fig. 8). Our microscopy, electroretinography and tracer-coupling experiments demonstrate that heterocellular gap junctions between AII and ON cone bipolar cells in the KO-Cx36-EGFP retina are normal, and similar to the WT, but homocellular AII–AII gap junctions are lacking. Thus, the mechanism(s) responsible for assembling Cx36–EGFP into heterocellular gap junctions in the KO-Cx36-EGFP retina are functional, whereas the mechanism(s) necessary for forming the homocellular synapse are non-functional in this genotype (Fig. 8). Therefore, we conclude that at least two distinct mechanisms for assembling Cx36 exist and can be simultaneously active in a single neuron.

Connexins are assembled into hexameric channels (connexons) before delivery to the membrane and assembly into gap junction channels (Chai et al., 2011). Some connexins – e.g. Cx30.3 – require heteromerization with another connexin (Plantard et al., 2003), whereas others – e.g. Cx50 – need specific interaction partners, such as the scaffold protein ZO-1 (Chai et al., 2011), to assemble into functional gap junctions.

For assembly into gap junctions in the olfactory bulb and cerebellum, the Cx36–EGFP fusion protein needs to heteromerize with wild-type Cx36 (Helbig et al., 2010) (Fig. 1). This requirement was attributed to an essential function of the C-terminal residues of Cx36, which are masked by EGFP in the fusion protein; a similar finding for the assembly of Cx50 *in vivo* was also recently demonstrated (Chai et al., 2011). We considered whether Cx45, also expressed at high levels in the ON IPL (Hilgen et al., 2011), might play a role in forming heterocellular junctions between AII and ON cone bipolar cells in the KO-Cx36-EGFP retina. Because Cx45 was shown to be coexpressed with Cx36 in the IPL (Li et al., 2008), it is tempting to speculate that the formation of a Cx36–EGFP–Cx45 heteromeric complex is necessary to incorporate Cx36–EGFP into heterocellular gap junctions in the KO-Cx36-EGFP retina. However, heteromeric connexin complexes would be present in all AII cells and the selective absence of such complexes from gap junctions between two AII cells cannot be rationalized without invoking additional mechanisms. The same holds true for a bihomotypic Cx36–Cx45 gap junction, as suggested by Li and colleagues (Li et al., 2008). A third possibility for connexin composition influencing the formation of heterocellular gap junctions arises from studies claiming that Cx45 is excluded from AII, but expressed in ON cone bipolar cells where it forms a heterotypic gap junction with AII-expressed Cx36 (Dedek et al., 2006; Maxeiner et al., 2005). Thus, one could suggest that a bipolar-cell Cx45-connexon could serve as a substrate for the addition of a Cx36–EGFP-containing connexon originating from an opposing AII cell; such a complex would be excluded from the AII–AII junction as Cx45 is not expressed in AII cells (Dedek et al., 2006). However, several observations argue against a role for Cx45: first, Cx36 is known to be expressed in ON cone bipolar subtypes (Deans et al., 2002; Han and Massey, 2005; Lin et al., 2005); at least one ON cone bipolar subtype (subtype 7) comprising about 25% of the total population was shown to contain Cx36 but lack Cx45 (Han and Massey, 2005; Lin et al., 2005). The presence of glycine in an equal number of ON cone bipolar cells in Cx36-containing and Cx36-lacking transgenic mice (Fig. 2A,C) indicates that this bipolar subtype is also able to form heterocellular gap junctions, which, in the KO-Cx36-EGFP mouse, would be expected to lack both wild-type Cx36 and Cx45. Second, experiments have demonstrated that a HeLa cell coexpressing Cx36 and Cx45 is able to colocalize the two connexins but that expressing single connexins in neighboring cells does not mediate colocalization (Li et al., 2008). Thus, the possible presence of Cx45 at the junction is not sufficient to drive assembly of Cx36 in the opposing cell. Finally, of note, neither of the scenarios outlined above are supported by our analyses using immunostaining (Fig. 7). The formation of heteromeric, bihomotypic or heterotypic Cx36–EGFP–Cx45 complexes should lead to a high degree of colocalization in confocal images of the KO-Cx36-EGFP retina, which, based on the number of Cx36–EGFP clusters located on dye-injected AII cells, is expected to be 55%. However, the staining shows a much smaller overlap (12%), demonstrating that Cx45 is not necessary for the incorporation of Cx36–EGFP into heterocellular junctions.

Although confocal microscopy is limited by light-resolution, the changes in fluorescence colocalization are consistent with only a limited role, if any, for ZO-1, in which ZO-1 interacts with the C-termini of Cx36 and Cx45 (Li et al., 2004b; Li et al., 2008), in the assembly of Cx36–EGFP at heterocellular gap junctions in KO-Cx36-EGFP mice. ZO-1 colocalizes extensively with Cx36–EGFP puncta in WT- and KO-Cx36-EGFP retinas, which supports an essential role for the protein in the formation of gap junctions. However, because the majority of gap junctions that colocalize with ZO-1 are not retained in the KO-Cx36-EGFP retina, we conclude that interaction with ZO-1 alone does not allow formation of gap junctions when the wild-type Cx36 protein is absent. Other scaffold and adhesion proteins colocalizing with Cx36 in the retina are present in the IPL (Ciolofan et al., 2006; Li et al., 2012a; Lynn et al., 2012) and might play a role in the specific targeting and assembly of Cx36-containing complexes. Immunostaining experiments for the effector and scaffolding proteins AF6 and MUPP1, which were shown to interact with Cx36 in the IPL (Li et al., 2012a), similarly exclude these proteins from playing a role in favoring the assembly of Cx36–EGFP at one gap junction over the other (supplementary material Fig. S1). However, a conclusive assessment of the involvement of ZO-1 and other protein candidates in discriminatory assembly of Cx36 into AII gap junctions could require other approaches. We note that the structural dissimilarity between the heterocellular and homocellular synapse, which is conspicuous by electron microscopy (Anderson et al., 2011; Kolb, 1979; Strettoi et al., 1992), could provide a visual clue to the discriminatory mechanisms and could be exploited to identify and/or localize pertinent molecules.

Based on the above observations, we suggest that the formation of homocellular or heterocellular gap junctions in AII cells involve Cx36 carboxy-terminus-dependent and -independent pathways, respectively; the former, in common with gap junctions between the neurons of the olfactory bulb and cerebellum, requires expression of genomic Cx36 for the assembly of Cx36–EGFP, whereas the latter utilizes a different mechanism entirely. ERG recordings and coupling experiments show that only heterocellular gap junctions are retained and functional in KO-Cx36-EGFP mice and indicate that this retention is nearly complete. Therefore, we think it unlikely that the presence of heterocellular gap junctions reflects some vague property of a defective molecule, or a redundant or abnormal assembly pathway, but argue that the artificially modified form of Cx36 has been able to highlight intrinsic differences in gap-junction-forming mechanisms, resulting in the selective retention of one of two gap-junction-classes in the AII cell. We have determined that the specific retention of heterocellular gap junctions in KO-Cx36-EGFP does not reflect a general difference in gap-junction-forming processes between axons and dendrites. The possibility that axon-specific mechanisms in axons of bipolar cells enable incorporation of Cx36–EGFP on both sides of the synapse in AII and ON cone bipolar cells, by capturing Cx36–EGFP connexons diffusing randomly in the dendrites of AII cells, can be refuted because gap junctions formed from Cx36–EGFP on CaB5-containing dendrites of bipolar cells are abundant in the OPL (supplementary material Fig. S2; Feigenspan et al., 2004). Alternative mechanisms are clearly not uncommon or restricted to the AII cell as Cx36–EGFP clusters are present in the outer retina and spinal cord, and we determined that ∼50% of all Cx36–EGFP clusters retained in the KO-Cx36-EGFP ON IPL are contributed by other amacrine and/or ganglion cells. Whether a single, common, mechanism is employed in the formation of all the above gap junctions remains to be determined.

### Functional relevance for multiple intracellular gap-junction-forming pathways

Previous studies have demonstrated that homocellular and heterocellular gap junctions perform different functions: AII–AII gap junctions serve signal averaging and noise reduction (Dunn et al., 2006), gap junctions between AII and ON cone bipolar cells are obligatory elements of the primary rod pathway (Bloomfield and Völgyi, 2009) with signal flow from AII to ON cone bipolar cells, and an approach-sensitive pathway (Münch et al., 2009) with signal flow from ON cone bipolar to AII cells. Moreover, the two types of junctions are regulated differently; AII–AII gap junctions by dopamine and cyclic adenosine monophosphate, and gap junctions between AII and ON cone bipolar cells by nitric oxide and cyclic guanosine monophosphate (Mills and Massey, 1995). They also exhibit ultrastructural differences (Anderson et al., 2011; Kolb, 1979; Strettoi et al., 1992). We now show that the mechanisms leading to their formation are also different. The importance of having different mechanisms for Cx36 assembly at the two gap junctions is unclear. However, Flores and colleagues (Flores et al., 2012) suggest that trafficking of Cx36 is important in regulating the strength of transmission at electrical synapses. Thus, different assembly mechanisms might allow the AII cell to alter the number of channels independently at its two sets of gap junctions; thereby distinctly altering synaptic strength. In addition to posttranslational modification of Cx36 (Kothmann et al., 2009), this strategy might underlie the differential light-dependent regulation of gap junctions between AII cells and those between AII and ON bipolar cells (Bloomfield and Völgyi, 2009) and contribute to the ability of the AII cell to process different visual streams in the retina.

## MATERIALS AND METHODS

Unless stated otherwise, all chemicals were purchased from Carl Roth GmbH (Karlsruhe, Germany).

### Animals and tissue preparation

All experiments were performed in accordance with institutional and national guidelines.

Transgenic mice expressing the Cx36–EGFP transgene (Feigenspan et al., 2004; Helbig et al., 2010) were backcrossed to Cx36-knockout animals (Deans et al., 2002) to generate KO-Cx36-EGFP mice. Puncta density in the ON IPL of HET-Cx36-EGFP (0.179/µm^2^±0.017; *n* = 18 scans, three mice) and WT-Cx36-EGFP mice (0.182/µm^2^±0.017; *n* = 18 scans, three mice) were similar (*P* = 0.907), and so the genotypes were used intermittently and designated HET-Cx36-EGFP. We specify WT-Cx36-EGFP only when all the animals used in the experiment had that genotype. WT (*Cx36*^+/+^) and KO (*Cx36*^−/−^) mice were used in control experiments.

Mice (aged 2–6 months) were deeply anesthetized with CO_2_ and euthanised by cervical dislocation. Eyes were enucleated, and cornea, lens and vitreous body were removed and the retina was isolated in Ringer's solution (110 mM NaCl, 2.5 mM KCl, 1 mM CaCl_2_, 1.6 mM MgCl_2_, 10 mM D-glucose, 22 mM NaHCO_3_, pH 7.4) and maintained by aerating with mixed gas (95% O_2_ and 5% CO_2_) for dye-coupling experiments and staining for glycine. For dye injections, a solution of 137 mM NaCl, 5.4 mM KCl, 1.8 mM CaCl_2_, 1 mM MgCl_2_, 10 mM D-glucose and 5 mM HEPES, pH 7.4 was used.

### Dye and tracer injections

For dye injections (Fig. 1), retinas were isolated from the eyecup, bisected and embedded in 2% agar-agar in Ringer's solution. Vertical sections (200 µm) were prepared as described previously (Dedek et al., 2006). AII amacrine cells were injected, under visual control, with microelectrodes (120–220 MΩ) filled with 7.5 mM Alexa-Fluor-594 potassium hydrazide (Invitrogen, Karlsruhe, Germany) diluted in 0.2 M KCl, pH 7.4. Cells were filled for 3–6 min. Tracer injections and whole-mount dye injections (Figs 4–6) were performed as described previously (Dedek et al., 2006; Shelley et al., 2006) using light-adapted mice. Retinal quarters were mounted, ganglion-cell side up, on black filter paper and incubated for 20 min in 0.1 mM DAPI diluted in Ringer's solution to visualize the nuclei of AII cells. Microelectrodes (120–180 MΩ resistance) were filled with 5 mM Alexa Fluor 488 and 4% (w/v) neurobiotin (Vector Laboratories, Burlingame, CA) or 7.5 mM Alexa Fluor 555 or 594. The Alexa dye was iontophoresed with 0.5 nA square pulses of 750 ms at 1 Hz for 1–2 min to visualize morphology; the current was reversed to inject positively charged neurobiotin (6–7 min), which was visualized with DyLight549-conjugated streptavidin (Jackson ImmunoResearch, West Grove, PA).

### Immunohistochemistry

Eyecups (2%), spinal cord (4%) and brain (4%) were fixed in paraformaldehyde and cryoprotected in 30% sucrose overnight. Cryosections (20–25 µm) were blocked with CTA [5% ChemiBLOCKER (Millipore, Billerica, MA), 0.5% Triton X-100 and 0.05% NaN_3_] or 5% donkey serum and then incubated with primary antibodies (supplementary material Table S1) or the nuclear dye TO-PRO3 (1∶500; Invitrogen). Whole-mount retinas for glycine and VGluT1 stainings were incubated in primary antibody for 5 days at 4°C and, after extensive washing, with secondary antibodies overnight. Cx36–EGFP puncta were not enhanced with antibodies. Specificity of the antibody against Cx45 (Invitrogen, 40-7000) (Li et al., 2008) was tested using a mouse line expressing a fusion protein of Cx45 and EGFP (Cx45–EGFP) (Hilgen et al., 2011). An almost perfect overlap between the antibody against Cx45 antibody and the Cx45–EGFP signal was observed.

### Fluorescence image acquisition and analysis

Retinas from the same experimental group were prepared, incubated and scanned with a Leica TCS SP2 or SP5 (Leica, Nussloch, Germany) under identical conditions. Unless stated otherwise, images are presented either as maximum or sum projections of the collapsed confocal stack and adjusted for brightness and contrast for presentation purposes. For quantitative analyses, we used the ImageJ software (NIH, Bethesda, MD). Analyzed images were background-subtracted and noise-reduced when relevant, and intensity and size thresholds were either set automatically or adjusted manually to best reflect visible clusters; threshold settings were kept constant for a particular experimental group. Unless stated otherwise, 12 independent scanned images were evaluated per mouse.

To evaluate densities, clusters were counted using the ‘analyze particle’ plugin in ImageJ and colocalization analysis was performed using the ‘colocalization highlighter’; three-color colocalization was carried out sequentially. Binary images of colocalized pixels are displayed (Fig. 5C,D; Fig. 7C,F,I,L) or shown as dots representing their centroids (Fig. 6A,B) in some of the figures. For determining Cx36–EGFP cluster size, colocalized clusters were identified as above and their size was determined in Metamorph 4D viewer (Molecular Devices, Sunnyvale, CA). The density of glycine-positive cells was determined from confocal stacks comprising the entire INL of glycine-immunolabeled whole-mount retinas (two mice per genotype). The ‘cell counter’ plugin was used to determine glycine-positive cell numbers from substacks comprising the proximal INL for amacrine cells or distal INL for bipolar cells.

The dendritic areas of the AII cells injected with the tracer were measured by drawing a convex polygon around the outermost tips of the dendrites, as revealed in a flattened stack. The average area for all injected AII cells was then used to define a circle for the plots in Fig. 5. For the distributions of coupled AII and ON cone bipolar cells relative to the injected AII cell for all WT and KO-Cx36-EGFP tracer-injection experiments, the *x*,*y* positions of the soma center (injected-AII and coupled cells) were determined from confocal stacks. The area of all tracer-coupled AII somata was defined as a circle whose radius was the distance from the farthest tracer-coupled cell to the injected AII. Similarly for cone bipolar cells, the centroid of all tracer-coupled cone bipolar cells was determined and the area of tracer-coupled cone bipolar somata was defined as a circle whose radius is the distance of the farthest cell from the respective centroid. This correction was necessary as the area of coupled cone bipolar cells was not always centered on the injected AII cell (for example, see Fig. 4I). The distances of all cone bipolar cells from the centroid are plotted (Fig. 5G). Although the plot retains the relative location of coupled cone bipolar cells among each other, it randomly allocates the different sets of coupled cone bipolar cells around the center of the injected AII.

### Electroretinography

Scotopic ERGs at ten different light intensities ranging from −3.5 to 1 log cds/m^2^ were recorded from ten mice of each genotype, as described previously (Kranz et al., 2013). Data analysis was performed using Chart v5.5 (AD Instruments, Hastings, UK).

### Statistics

Statistical tests were performed with GraphPad Prism 5 (GraphPad Software, San Diego, CA) or MatLab (MathWorks, Natick, MA) or Excel (Microsoft Corporation, Redmond, WA). Values are presented as mean ± standard error of the mean (s.e.m.). Data were subjected to the D'Agostino and Pearson, Shapiro-Wilk, or Kolmogorov–Smirnov tests for determining normality and tested for significance with an unpaired two-tailed *t*-test, ANOVA or the Mann–Whitney U-test. Pair-wise comparisons were conducted using post hoc Bonferroni tests.

## Supplementary Material

Supplementary Material
