# Supplementary material for: AII amacrine cells discriminate between heterocellular and homocellular locations when assembling connexin36-containing gap junctions
Source: J Cell Sci. 2014 Mar 15;127(6):1190–202. doi: 10.1242/jcs.133066 (PMC3953814; doi:10.1242/jcs.133066)
Supplement: Supplementary Material [file supp_127_6_1190__index.html]

AII amacrine cells discriminate between heterocellular and homocellular locations when assembling connexin36-containing gap junctions — Supplementary Material 

# AII amacrine cells discriminate between heterocellular and homocellular locations when assembling connexin36-containing gap junctions

## JCS133066 Supplementary Material

**Files in this Data Supplement:**

- **Supplementary Material**
