# Supplementary material for: AII amacrine cells discriminate between heterocellular and homocellular locations when assembling connexin36-containing gap junctions
Source: J Cell Sci. 2014 Mar 15;127(6):1190–202. doi: 10.1242/jcs.133066 (PMC3953814; doi:10.1242/jcs.133066)
Supplement: Supplementary Material [file supp_127.6.1190_JCS133066.pdf]

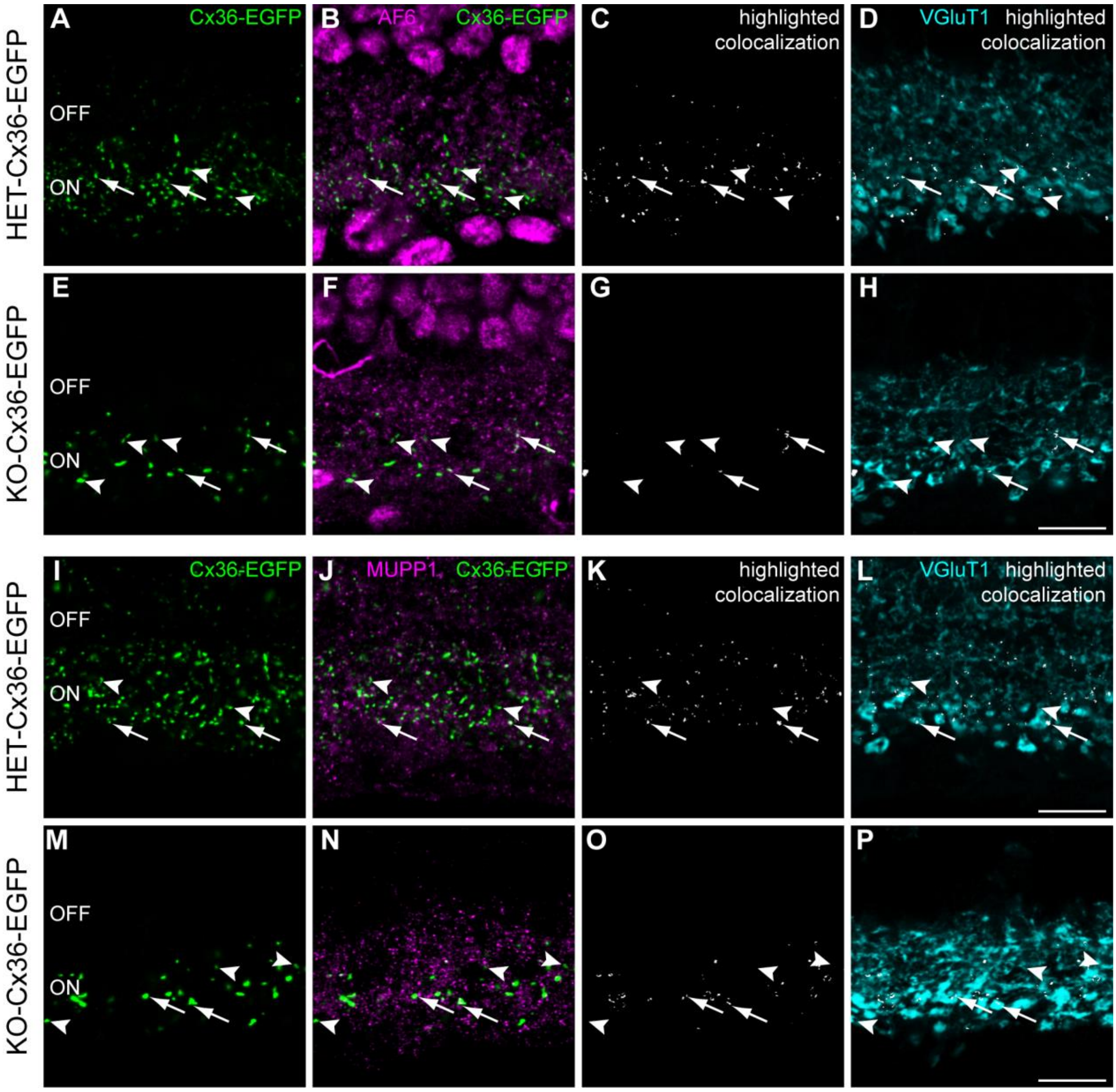

Figure S1. The exclusive assembly of Cx36-EGFP into AII–ON CB gap junctions in the KO-Cx36- EGFP retina is not mediated by a possible association with AF6 or MUPP1. Colocalization of Cx36-EGFP with AF6 and MUPP1 in HET-Cx36-EGFP (A-D, I-L) and KO-Cx36-EGFP mice (E-H, M-P). Cx36-EGFP puncta (green; A,B,E,F,I,J,M,N) colocalized with AF6 (magenta; B,F,) and MUPP1 (magenta; J,N). Colocalizing clusters are highlighted in C, G, and K, O, respectively, and are projected onto VGluT1-labeled bipolar cell terminals (cyan; D,H,L,P). Arrows indicate Cx36-EGFP puncta colocalizing with AF6 or MUPP1 that are located on bipolar cell terminals. Arrowheads point to Cx36-EGFP puncta located on bipolar cell terminals that do not colocalize with AF6 or MUPP1. The presence of clusters of the latter class (arrowheads) argue against an obligatory involvement of the two proteins in Cx36-EGFP assembly, which is also supported by the fact that the number of clusters colocalizing with each of the two proteins in the HET-Cx36-EGFP retina is larger than the number of clusters retained in the KO-Cx36- EGFP retina. Scale = 10 μm.

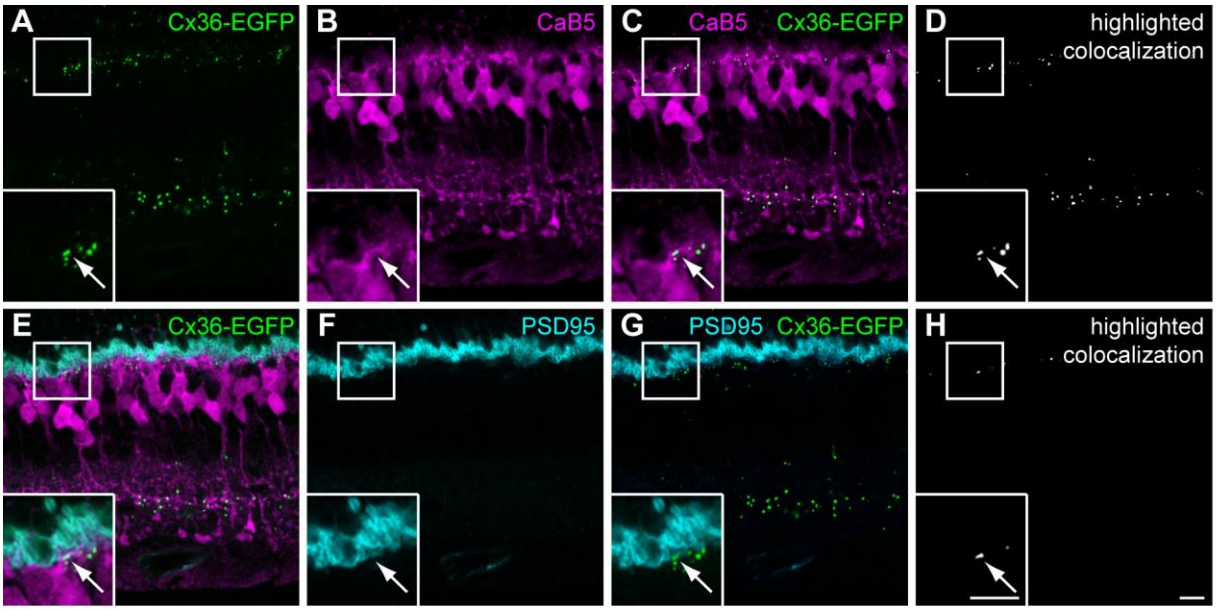

Figure S2. In the outer plexiform layer of KO-Cx36-EGFP mice, Cx36-EGFP is localized on bipolar cell dendrites and photoreceptor terminals. Vertical section of the KO-Cx36-EGFP retina labeled for calcium binding protein 5 (CaB5, a bipolar cell marker; magenta; B,C,E) and PSD95 (a marker for photoreceptor terminals; cyan; E-G). Cx36-EGFP clusters (A) are colocalized with CaB5-positive bipolar cell dendrites (C,E) and PSD95-stained photoreceptor terminals (G,E) as illustrated in D and H, respectively, which highlight the colocalized pixels (arrows point to colocalized clusters in insets). Areas marked by squares are shown in higher magnification in the insets. The presence of numerous Cx36-EGFP clusters that are present on bipolar cell dendrites (C,D) suggest that Cx36-EGFP may also be incorporated into dendrites. This is inconsistent with the hypothesis that AII-ON CB gap junctions only form because there are different gap junction-forming mechanisms in axons and dendrites. Scale = 10 μm.

Table S1: Primary antibodies used.

Antibody	Host	Source	Catalog No	Dilution
anti-AF6	rabbit	Invitrogen	700193	1:500
anti-CaB5	rabbit	kind gift of Francoise Haeseler, University of Washington, Seattle, USA		1:1,000
anti-calbindin	rabbit	Swant	CB-38a	1:1,000
anti-Cx36	mouse	Invitrogen	37-4600	1:500
anti-Cx45	rabbit	Invitrogen	40-7000	1:1,000
anti-glycine	rat	kind gift of David Pow, University of Queensland, Brisbane, Australia		1:1,000
anti-MUPP1	rabbit	Invitrogen	42-2700	1:500
anti-VGluT1	guinea pig	Millipore	AB 5905	1:1,000 – 1:2,000
anti-ZNP-1 (Syt2)	mouse	Zebrafish International Resource	Zmol81503	1:500
anti-ZO-1	mouse	Invitrogen	339100	1:250
